# Recent Advances in Nanoparticle-Based Co-Delivery Systems for Cancer Therapy

**DOI:** 10.3390/nano12152672

**Published:** 2022-08-04

**Authors:** Rouba D. Al Bostami, Waad H. Abuwatfa, Ghaleb A. Husseini

**Affiliations:** 1 Department of Chemical Engineering, College of Engineering, American University of Sharjah, Sharjah P.O. Box 26666, United Arab Emirates; 2 Materials Science and Engineering Program, College of Arts and Sciences, American University of Sharjah, Sharjah P.O. Box 26666, United Arab Emirates

**Keywords:** co-delivery, multi-drug resistance, nanocarriers, gene delivery

## Abstract

Cancer therapies have advanced tremendously throughout the last decade, yet multiple factors still hinder the success of the different cancer therapeutics. The traditional therapeutic approach has been proven insufficient and lacking in the suppression of tumor growth. The simultaneous delivery of multiple small-molecule chemotherapeutic drugs and genes improves the effectiveness of each treatment, thus optimizing efficacy and improving synergistic effects. Nanomedicines integrating inorganic, lipid, and polymeric-based nanoparticles have been designed to regulate the spatiotemporal release of the encapsulated drugs. Multidrug-loaded nanocarriers are a potential strategy to fight cancer and the incorporation of co-delivery systems as a feasible treatment method has projected synergistic benefits and limited undesirable effects. Moreover, the development of co-delivery systems for maximum therapeutic impact necessitates better knowledge of the appropriate therapeutic agent ratio as well as the inherent heterogeneity of the cancer cells. Co-delivery systems can simplify clinical processes and increase patient quality of life, even though such systems are more difficult to prepare than single drug delivery systems. This review highlights the progress attained in the development and design of nano carrier-based co-delivery systems and discusses the limitations, challenges, and future perspectives in the design and fabrication of co-delivery systems.

## 1. Introduction

Cancer is one of the most pervasive diseases and is classified as the second leading cause of death worldwide, after cardiovascular diseases. According to the World Health Organization (WHO) [1], approximately 4807 new cancer cases were reported in 2020 in the United Arab Emirates (UAE), with 1896 deaths and 14,816 prevalent cases, i.e., cases lasting more than 5 years [2,3]. Cancers, also known as malignant neoplasms, are a group of diseases discerned as the uninhibited growth and multiplication of abnormal cells at any site in the body. Recently, there have been countless advancements in developing effective cancer treatments and therapies. Advancements in modern anti-cancer therapies, such as gene therapy, have also achieved remarkable results in synergistic anti-tumor effects. Likewise, immunotherapy is also considered a promising therapeutic platform [4], where researchers manipulate the body’s immune system to hinder the haphazard division and spread of cancer cells through the bloodstream and into adjacent organs and other tissues [5]. Despite the latter advances, cancer treatment continues to be strenuous and challenging due to the complexity of cancer, and hence conventional chemotherapy remains the fundamental therapeutic approach [4,5,6]. Chemotherapy, defined as the systemic administration of cytotoxic agents that halt the division and proliferation of cancer cells, has several side effects that limit its effectiveness [6], including (i) low solubility and bioavailability, that often results in irregular biodistribution, causing poor localization of the chemotherapeutic agent at the tumor site, (ii) detrimental systemic toxicity, that affects normal cells and healthy tissues [7], and (iii) multi-drug resistance (MDR) induced by long-term and continuous administration, which is considered a critical consequence [5,6,8,9].

MDR hampers the formulation of efficient and long-lasting cancer treatments. MDR emerges rapidly throughout, or following, the treatment and can lead to cross-resistance against additional distinct chemotherapeutic agents with different structures, functions, and/or targets. MDR can be attributed to various mechanisms, including the alteration of the metabolic pathways of drug action in cancer cells and the acclimatization of the cancer cells to the microenvironment. These mechanisms can act simultaneously, therefore reducing intracellular drug concentrations in cancer cells through lowering absorption and boosting efflux [10]. In addition, MDR can also be attributed to the overexpression of ABC transporters in cancer cells, such as ABCB1 (also known as P-glycoprotein (P-gp)). ABC transporters are proteins bound to the cell membrane that utilize ATP to transport their substrates, such as carbohydrates, proteins, amino acids, lipids, peptides, and many more building blocks, through the cell membrane [11]. The overexpression of ABC transporters decreases the cells’ sensitization to chemotherapeutic drugs, hence, changing the drug’s metabolic route, actuating DNA restoration function, etc. Consequently, MDR is a crucial factor that annuls the effect and development of a wide scope of chemotherapeutics agents [12,13,14,15]. Other studied predictive markers that confer MDR include the differential overexpression of SEMA6D protein, liver x receptor alpha (LXRα) transcription factors, and ITGA7 genes in breast cancer cells [16,17,18].

Often, the use of single-drug chemotherapy, also known as monotherapy, is insufficient to provide a comprehensive therapeutic plan that is effective in treating cancer [19]. As a result, attention has been directed toward designing drug delivery systems to co-deliver several combinations of therapeutic agents to overcome the drawbacks of single-drug chemotherapy [20,21]. Some of these drawbacks include low response rates, inefficiency in completing tumor treatment, and drug resistance. These drawbacks result in tumor reappearance and aggressive proliferation, in addition to widespread distribution and the hindering of only limited etiopathogenic pathways. Carrick et al. conducted a detailed study to compare single-drug chemotherapy against combination chemotherapy for metastatic breast cancer [22]. The study showed that women receiving combination chemotherapy treatments showed statistical improvement in tumor regression and progression rates; however, high toxicity was detected. More research needs to be done to ascertain whether administering single-drug chemotherapeutics sequentially would be more efficient than combination therapy.

The concept of the co-delivery approach first occurred from merging two research fields: drug delivery and gene therapy. Co-delivery systems combine at least two therapeutic agents with different physiological and physicochemical properties; thus, achieving clinical combination chemotherapy [5,12,23]. Taking into consideration the co-delivery strategy, combination therapy via nanotechnology approaches has progressively become a desirable technique and one of the leading frontiers in research to find an efficient drug delivery system (DDS) [23,24]. There is a substantial requirement for the development and progression of innovative techniques to treat cancer using DDS systems. Hence, there has been a surge in academic publications on co-delivery systems ascribed to the synergistic and undisruptive procedures these systems convey in treating diverse types of cancer [23,25]. To accomplish a guaranteed and efficient, targeted delivery of therapeutic drugs, an extensive interpretation of the interaction mechanism between the cancer cells and the DDS is essential [14,26]. The scope of this review was to address recent progress in the field of nanotechnology for combination therapy used in co-delivery systems. Additionally, this review discusses the relevance of combination therapy, as well as the limitations, challenges, and future perspectives in designing and assembling co-delivery systems.

## 2. Nanocarriers Used in Co-Delivery Systems

Several commonly studied nanocarriers can be used to construct a co-delivery system that enables a synergistic effect between two or more medications [15]. The clinical treatment of cancer has utilized more than 20 nanotechnology-based therapeutic products [26]. With progression in the field of nanomaterials and nanotechnology, researchers have worked intensively to build multi-functional nanocarriers that can meticulously transport therapeutic agents to tumor sites [27,28,29]. Nanoparticles are being extensively used as diminutive drug delivery vehicles to amplify the advantages of a co-delivery system. In general, the possible co-delivery approaches using different types of nanocarriers have proved to be efficient in repressing MDR, improving efficacy and drug targeting, achieving a synergistic therapeutic effect, and reducing systemic toxicity and the adverse effects of chemotherapeutic agents [23,25,28,30].

Nanocarriers possess unique characteristics, such as a nanometric size, a high surface area to volume ratio, advantageous drug release patterns, and targeting qualities that can help them accumulate preferentially in tumor tissues [15]. Most importantly, nanomaterials can significantly increase the accumulation of their load at tumor locations by utilizing the enhanced permeability and retention effect (EPR) [4,30]. EPR is a phenomenon that is hypothesized to be caused by leaky tumor vasculature and poor lymphatic outflow. EPR is crucial in the realm of nanotechnology, specifically the passive targeting of nanoparticles in the tumor microenvironment, and it has been explored extensively by Galiardi et al. [13,25,27]. The development of more effective nanoparticles to deliver desired multiple payloads concurrently is a major challenge. Multiple biological parameters, such as interaction with plasma proteins, blood circulation time, extravasation, tumor tissue penetration, and cancer cell uptake, might alter the biodistribution of systemically administered nanoparticles [24]. Surface modification of nano-systems that bestow unique targeting capabilities or stimuli-sensitive responses impact the nanoparticles’ overall in vivo behavior [30,31]. Much of our present understanding of nanoparticle systems is based on in vitro studies and on animal models for in vivo data [25,30,32,33].

**Figure 1:**
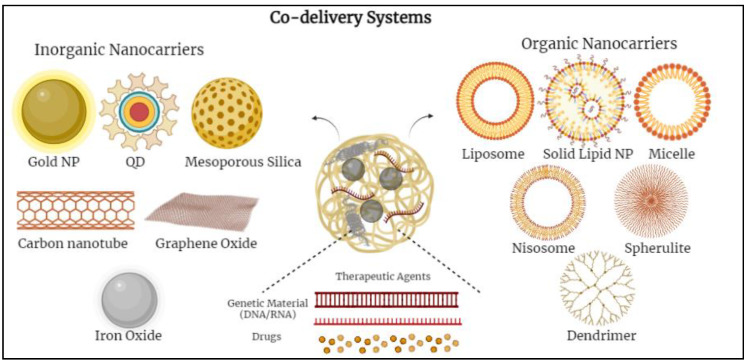
Schematic diagram demonstrating various co-delivery systems and the different inorganic- and organic-based nanocarriers.

Various nanoparticles (NPs) have been examined to design novel co-delivery systems. There are two main groups of nanocarriers divided into inorganic-based and organic-based nanoparticles. Inorganic-based nanoparticles mostly include mesoporous silica nanoparticles, iron oxide nanoparticles, gold nanoparticles, quantum dots, etc.; whereas, organic-based nanoparticles include polymeric micelles, nano-emulsions, polymersomes, liposomes, dendrimers, noisomes, etc. [12,14,34] (Figure 1).

### 2.1. Inorganic-Based Nanoparticles

Various inorganic nanoparticles have been developed, such as mesoporous silica nanoparticles (MSNs), gold nanoparticles (Au NPs), quantum dots, and iron-based nanoparticles. Using inorganic nanoparticles as co-delivery carriers frequently necessitates nanoparticle modification [4,13]. To accomplish an efficient and successful co-delivery, two or more physical or chemical modifications can be used. Even though inorganic nanoparticles have received a lot of interest as potential delivery vesicles, the FDA (Food and Drug Administration) and the EMA (European Medicines Agency) have only approved NanoThermVR as a drug delivery system (DDS) for limited anti-cancer therapy for glioblastoma, prostate, and pancreatic cancer [13,35]. None of the inorganic nanoparticles for co-delivery have been approved to be available on the market as they have not yet crossed the first clinical stages. Pegylated colloidal gold nanoparticles conjugated to tumor necrosis factor-alpha (TNFa) particles for cancer therapy and silicon nanocarriers for parenteral peptide administration, for example, are in early clinical development [4,32]. At the moment, gold and mesoporous silica nanoparticles are receiving the greatest attention in terms of experimentation and development [31].

#### 2.1.1. Gold Nanoparticles (Au NPs)

Au NPs are considered an important breakthrough and have shown considerable potential in the delivery of chemotherapeutic agents for cancer treatment. The biocompatibility, adaptability, and non-immunogenic properties of Au NPs have allowed their incorporation into various treatments, such as radio-sensitizers, photothermal agents, and drug delivery vehicles [31]. Xiao et al. co-delivered the chemotherapeutic drug doxorubicin (DOX) and small interfering RNA (siRNA) against the achaete-scute complex-like 1 (ASCL1) gene, particularly to neuroendocrine cancer cells [35]. According to literature, ASCL1 is one of the most extensively expressed genes, and is involved in cell division and multiple other cellular functions, including intracellular transportation. ASCL1 protein expression in the body, and its role as a possible oncogene, are critical in the growth and advancement of several types and forms of cancer [36,37]. For targeting tumor cells specifically, the exterior surface of Au NPs can be altered by active targeting ligands or moieties. Au NPs passively accrue and retain preferentially in tumor locations because of the EPR effect [31,38]. Therefore, to also benefit from the acidic tumor microenvironment, a pH-sensitive multi-functional gold (Au) nanorod-based nanocarrier with distinctive properties was conjugated with octreotide (OCT) [4,32,36]. Octreotide is a cancer-targeting ligand, that attaches to overexpressed somatostatin receptors in neuroendocrine (NE) cancer cells [38]. The Au-DOX-OCT complexed with ASCL1 siRNA showed much stronger gene silencing and anti-proliferative effects in NE cancer cells than non-targeted Au-DOX complexed with ASCL1 siRNA [5,13,37]. This study reported synergistic effects between the co-delivered DOX and siRNA in inhibiting cell growth and cancer suppression. As a result, targeting cancer cells through a combination of chemotherapy and siRNA-mediated gene silencing using gold nanorods as nanocarriers has a lot of promise for improving therapeutic results in neuroendocrine tumors [32,36,39,40]. Other metal-based nanoparticles, such as iron oxide nanoparticles, have also been explored and possess several unique qualities because of their metal bonding, minute size effect, and surface properties [40]. Iron oxide nanoparticles that are minute and superparamagnetic with a diameter of roughly 10 nm can be utilized in thermotherapy and actively targeted cancer treatment [5,39]. It should be emphasized that when particle size decreases, some metal-based nanoparticles might interact with antioxidant defense mechanisms, resulting in increased cytotoxicity [18,41].

#### 2.1.2. Mesoporous Silica Nanoparticles (MSNs)

Mesoporous silica nanoparticles (MSNs) are alternative key inorganic-based nanoparticles used in co-delivery. MSNs are optimal models for loading significant amounts of chemotherapeutic agents and pairing other components on the surface because of their high surface area to volume ratio and vast aperture volume [13,42,43]. The latter characteristics allow MSNs to be used in several cancer treatments. On the surface of MSNs, the chemical modification of a significant quantity of hydroxyl groups makes it easier for them to encapsulate medications or functionalize them later [35]. Meng et al. examined a co-delivery system that allocated the physical encapsulation of DOX molecules in the pores of MSNs, and microRNAs were conjugated onto the surface through disulfide bonds [5,15,43]. The experimental results depicted that, in vivo, the combination therapy exhibited improved therapeutic effectiveness and reduced systemic toxicity [28,43]. Several experimental studies showed some differences and variations between in vitro and in vivo outcomes. The MSNs induced endothelial injury and this damage appeared to coincide with thrombosis generated by MSN build-up and accrued in the tissues [43]. It is worth mentioning that by changing the nanoparticle surface, for example, by grafting poly(ethylene glycol), also known as PEGylation, the accumulation potential of silica nanoparticles may be considerably reduced [44]. Yiu et al. created magnetic core-encompassing MSNs covered with PEI (polyethyleneimine) for plasmid DNA (pDNA) transfer in vitro [45]. This nanoparticle design could also incorporate a targeting agent to offer selectivity in the tumor microenvironment where nucleic acids would get artificially introduced.

The PEI-coated MSN co-delivery system has gained significant acceptance among researchers; hence studies have already been conducted to analyze multiple drug delivery methods to enable synergistic therapeutic approaches [44,46,47]. Meng et al. created PEI-coated MSNs to transmit DOX and a siRNA that inhibits P-gp synthesis [47]. The study showed an increase in the cytotoxicity of the cancer cells combined with an MDR cell line using this dual delivery approach, compared to PEI-coated MSNs carrying only DOX [48,49]. Further studies showed that a refined methodology, delivering optimum proportions of P-gp –siRNA–DOX with PEGylated PEI-coated MSNs in an in vivo MDR breast cancer model, showed synergistic tumor growth reduction with considerable P-gp knockdown at heterogeneous tumor locations [50,51,52]. Wang et al. employed a technique quite similar to the technique Meng et al. used in treating squamous cell cancer (SCC) [47]. In this study, a significant reduction in tumor growth, ~80% decrease in 28 days, in vivo after the administration of PEI-coated MSNs to co-deliver DOX and siRNA against MDR1 (P-gp1) was observed [50,51,53].

#### 2.1.3. Copper Nanoparticles (Cu NPs)

The continuous desire for novel therapeutic agents has fueled metal-based chemotherapeutic research. Metal-based therapeutic agents have generated the possibility of less toxic action against cancer cells and have demonstrated anti-proliferative action against cancer cells [53]. Copper has captured the attention of many researchers among metal-based nanoparticles due to its strong redox potential and its use as an adaptable carrier for drug delivery and phototherapy. Copper nanoparticles can change the regulation of over 1000 genes, such as metallothionein, glutathione synthetase, chaperones, histones, etc. [54,55]. Copper nanoparticles alter cell shape, reduce cell metabolic activity, raise oxidative stress, thereby causing mitochondrial damage, and eventually damage DNA by creating reactive oxygen species. Endocytosis is used for copper nanoparticle adsorption. An investigation of cancer morphology reveals that the production of copper nanoparticles dramatically increases cancer cell apoptosis; therefore, exerting an anti-cancer effect [55]. When chitosan is mixed with copper nanoparticles, the rate of cell death increases substantially. However, copper-based nanoparticles, including copper oxide (CuO), amorphous and crystalline copper sulphide (CuS), copper phosphate (CuPO_4_), and copper iodide (CuI), have been shown to be cytotoxic to human cancer cells [53].

Pramanik et al. recognized the capacity of copper nanoparticles as an anticancer drug and created a unique copper-based nanoparticle utilizing copper carbonate (CuCO_3_), which has efficient cytotoxic action [56]. Since CuCO_3_ nanoparticles are cytotoxic to normal cells, CuCO_3_ nanoparticles are conjugated with folic acid (FA-CuCO_3_) to reduce their cytotoxicity and directly target the tumor cells. CuCO_3_ nanoparticles caused DNA damage and disrupted the mitochondrial membrane, resulting in apoptosis-mediated cell death [57]. To test the targeting efficacy of CuCO_3_ nanoparticles, shRNA was used to create folate receptor knockdown HeLa cells. In animal models, Pramanik et al. reported that FA-CuCO_3_ nanoparticles were useful and had the potential to be an effective treatment agent for folate receptor-expressing cancer cells while minimizing cytotoxicity to normal cells [56]. Guo et al. utilized CuO nanoparticles as drug carriers for targeted drug delivery in nasopharyngeal cancer [58]. CuO nanoparticles were loaded with DOX and docetaxel, which are well-established chemotherapeutic drugs that have been used successfully for nasopharyngeal carcinoma. In order to reduce the cytotoxicity of CuO nanoparticles, Guo and co-workers coated CuO nanoparticles with varying amounts of PLGA (polylactide-co-glycolide) and then conjugated them with folic acid to improve tissue targeting [59,60]. The results of the study conveyed that the PLGA coating of CuO nanoparticles changed the surface characteristics of the nanoparticles and improved biodegradation of the nanoparticles within the body while causing no cytotoxicity to normal cells [59,61]. Copper nanoparticles, along with other metallic nanomaterials-based therapeutic treatments and agents, have exhibited considerable maximal efficacies in destroying cancer cells. Nevertheless, more extensive research and investigations are still needed on copper-based nanoparticles to gather accurate data on local toxicity and permanent side effects on the body [61]. Thus, significant preclinical and clinical trials are required before successful translation into clinical use and eventual acquisition of FDA approval [62,63].

### 2.2. Organic-Based Nanoparticles

A range of lipid-based carriers with varying physicochemical characteristics and structures, depending on the lipid content and manufacturing procedures, have been used. Most lipid nanocarriers are characterized by their biocompatibility, elevated drug loading competence, stability in vivo, and controlled drug release patterns [12,39]. Liposomes, niosomes, lipid micelles, nano-emulsions, solid lipid nanoparticles, polymeric nanoparticles, chitosan-based nanoparticles, and nanostructured lipid carriers are all examples of lipid-based nanocarriers [14,64,65]. However, the most eminent and significant lipid-based nanocarriers for efficient co-delivery are liposomes and nano-emulsions [14,66].

#### 2.2.1. Liposomes

Lipid-based nanostructures have been widely utilized in biological applications in the previous decade due to their biological consistency and compatibility. Lipid bilayer membranes assemble phospholipid carriers known as liposomes. Chemotherapeutic agents that are hydrophilic can be encapsulated in the inner aqueous phase, whereas hydrophobic drugs can be contained in the lipid layer [4,13,19]. Hydrophobic drugs have a low encapsulation rate because they are only entrapped in the bilayer interface. Liposomes are mostly made up of phospholipids generated by non-ionic surfactants and other amphipathic chemicals that permit self-assembly [25]. Liposomes are also made up of amphiphilic lipids that form spherical bilayers, similar to the lipid bilayer seen in biological membranes. The increased permeability and retention (EPR) effect generated by the specific anatomical and pathophysiological properties of tumor vasculature causes lipid nanocarriers to accumulate in tumor tissue passively [27,33].

**Figure 2:**
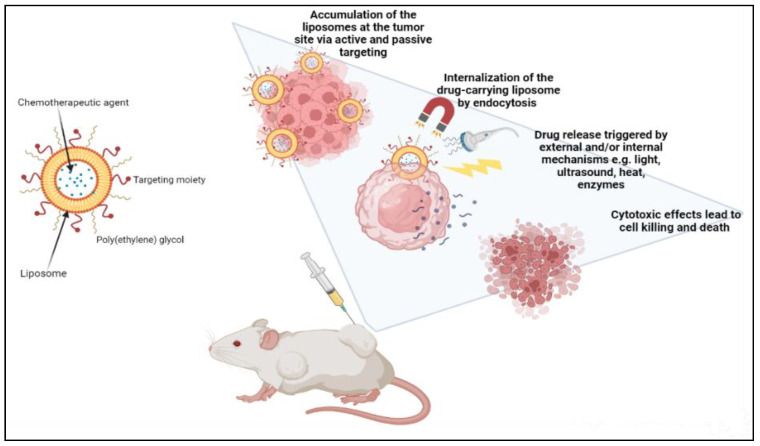
Demonstration of cancer treatment using a liposome-based drug delivery system.

Liposomes can be actively guided for identification by receptors at the tumor location by synthetically altering the liposome surface or terminal PEG (polyethylene glycol) molecule with tumor-specific ligands, such as folic acid, transferrin, or monoclonal antibodies. PEGylation shields the liposome nanocarrier from immune system destruction, thus, increasing the liposome circulation duration [30,33,64]. As a result, the liposome highly accumulates in tumor tissue while decreasing mononuclear phagocyte system absorption. The accumulation of the liposomes at the tumor site can occur either via active or passive targeting. An extra degree of complexity and specificity for the target cell may be accomplished via ligand-mediated targeting, also known as active targeting. After accumulating at the tumor site, drug-carrying liposomes are often taken up intracellularly by endocytosis. The process of endocytosis facilities the liposomes going through endosomes and lysosomes [25,32,39]. Hence, to release the drugs from the liposome intracellularly, the liposome membrane and the cancer cell must merge, or lipid mixing should occur with the endosomal or lysosomal membranes. Drug release can be triggered by external and/or internal mechanisms such as ultrasound, heat, enzymes, pH, etc. AlSawaftah et al. established that ultrasound, at a specific intensity and frequency, induces transient pores, also known as sonoporation, which enhances drug release from the liposomes and drug uptake in cancer cells [66] (Figure 2).

Traditional doxorubicin liposomes, such as Evacet and Myocet, doxorubicin liposomes with prolonged circulation times like Doxil and caelyx, cytarabine liposomes like Depocyt, and paclitaxel liposomes, such as Taxo, are all available on the market [5,27]. Liu et al. created an efficient multilayer liposome vesicle that can prolong the release of DOX and PTX, improving the benefits of the co-delivery system while decreasing systemic toxicity [18]. Sheng et al. employed nanoliposomes to transport perfluorooctyl bromide and ICG for improved multimodal imaging-guided phototherapy [67]. Patel et al. used a thin film hydration approach to create furtive liposomes encapsulated with the P-gp inhibitor tariquidar and the paclitaxel (PTX) [13,32]. An experiment was performed on SKOV-3TR cells, cellosaurus cell line, where the concurrent administration of tariquidar and PTX through prolonged circulating liposomes caused more cytotoxicity in the cancerous cells than administering paclitaxel only, indicating a considerable reversal of the MDR about PTX [13,68,69]. Even though various liposome nano-systems have been presented, liposomes’ limited drug loading capacity and poor stability make them unsuitable for large-scale use [23,25,66,69].

#### 2.2.2. Lipid Nano-Emulsion (NE)

Nano-emulsion nanocarriers have been explored as an alternative to outweigh the problems with liposome encapsulation efficiency and solubility. Different delivery techniques using nano-emulsion carriers have been utilized as therapeutic vehicles for hydrophobic drugs. Nanodroplets are distributed in a continuous phase in nano-emulsions [18]. Nano-emulsions generally comprise heterogeneous non-miscible liquids sustained by an emulsifying agent or a non-ionic surfactant. Cancer therapy research has shifted towards nano-emulsions because of their crucial properties in attaining effective therapeutic effects [69]. Their high surface area, physical stability, amphiphilicity, longer circulation time, precise targeting, tumor imaging characteristics, and surface functionalization for passive or active targeting allow nano-emulsions to readily concentrate in tumor vasculature and pass through challenging barriers [70,71]. Liu et al. devised a nano-emulsion to enhance chemotherapy delivery and efficacy to lower MDR in nasopharyngeal cancer treatment as an alternative to lowering MDR [13,19,72]. Ultrasonic homogenization was used to make nano-emulsions that efficiently co-delivered DOX and a radiotherapeutic isotope of yttrium-90 (90Y) [14]. The latter system was examined in vivo and in vitro, resulting in a substantial decrease in nasopharyngeal cancer cells. Due to the simultaneous blockage of many pro-tumor pathways, the biological results of a modified nano-emulsion demonstrated a significant reduction in tumor volume in a nude mouse model and an increase in HepG-2 cell death [14,39,69]. Many lipid-based delivery methods have reached the clinical testing phase. Weissig et al. researched and experimented on the co-administration of cytarabine and daunorubicin, also referred to as CPX-351, in patients with acute myeloid leukemia. The latter also investigated CPX-1, which is the encapsulation of irinotecan and floxuridine, specifically in patients with colorectal cancer [71]. The administration of CPX-351 and CPX-1 showed a higher remission rate and a prolonged survival rate in patients. The latter are the two major arrays of liposomal formulations of small molecule drug combinations that have commenced clinical trials [73,74,75]. Table 1 summarizes various nanomedicines currently under clinical trials for cancer treatment.

Lipid nanocarriers are commonly employed in co-delivery systems because of their varied hydrophilicity and low cytotoxicity; nevertheless, they might be unstable in systematic circulation and have a limited half-life in the body [13,14]. Manufacturing hybrid nanoparticles that combine lipids with other materials, such as synthetic polymers or inorganic material, is another way to reduce colloidal instability [76,77].

**Table 1:** Nanomedicines undergoing clinical trials for cancer treatment.

Commercialized Formulation (Active Ingredient)	Nanocarrier Type	Indications	Company	Clinical Trial Phase	Reference
Onco-TCS (Vincristine)	Liposomes	Non-Hodgkin Lymphoma	INEX Pharmaceuticals	Clinical phase 1/2	[78]
OSI-211 (Lurtotecan)	Liposomes	Lung cancer Recurrent ovarian cancer	OSI	Clinical phase 2	[79]
LEP-ETU (Paclitaxel)	Liposomes	Ovarian, breast, and lung cancers	Neopharma	Clinical phase 1/2	[78]
Auroshell	Gold-silica nanoshells	AuroLase therapy for cancer	Nanospectra Biosciences	Clinical phase 1	[80]
Thermodox (Doxorubicin)	Liposomes	Hepatocellular carcinoma	Celsion	Clinical phase 3	[78]
Aroplatin (Cisplatin analog)	Liposomes	Colorectal cancer	Antigenics, Inc.	Clinical phase 1/2	[79]
Nektar-102 (PEGylated irinotecan)	Liposomes	Breast, colorectal cancer	Nektar therapeutics	Clinical phase 3	[78]
NKTR-105 (PEG-Docetaxel)	Polymer-drug conjugate	Solid tumors	Nektar therapeutics	Clinical phase 1	[80]
CYT-6091 Aurimmune (TNF-α)	TNF-α bound to colloidal gold nanoparticles	Head and neck cancer	Cytimmune Sciences	Clinical phase 2	[78]
Paclical (Paclitaxel)	Polymeric micelles	Ovarian cancer	Oasmia Pharmaceutical AB	Clinical phase 3	[79]
Lipoplatin (Cisplatin)	Liposomes	Pancreatic, head and neck, breast cancer	Regulon	Clinical phase 3	[78]

#### 2.2.3. Polymeric Micelles

Polymer-based delivery systems are one of the most common platforms for delivering chemotherapeutic agents in co-delivery systems [19]. Polymer-based nanocarriers that load and distribute anti-tumor agents have piqued attention and become a focus for research to get around the limitations of the single delivery of chemotherapeutic agents. Polymer-based delivery systems are novel drug delivery systems that are prominently stable and reliable alternatives to lipid-based carriers [8,21]. Natural and synthetic polymers have been used to create polymeric nanocarriers [26]. Polymer-based micelles, polymersomes, polymer nanoparticles (NPs), nanogels, and mixed polymeric nanoparticles with porous cores are the five main forms of polymer nanocarriers [21,24].

**Figure 3:**
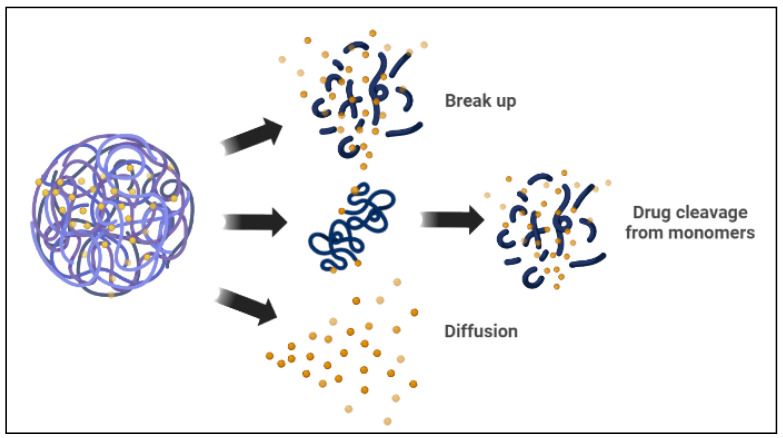
Drug release mechanisms from polymeric micelles.

Polymeric micelles are spherical amphiphilic, biodegradable, and flexible nanoparticles made by combining various amphiphilic di-block or tri-block copolymers. In an aqueous medium, the polymeric micelles have hydrophilic and hydrophobic blocks [80]. The hydrophobic micelle cores serve as a reservoir for hydrophobic drug encapsulation, whereas the hydrophilic shells can capture hydrophilic molecules [15,33]. There are two basic mechanisms the polymeric micelle follows to release the encapsulated drugs. The micelle dissociates, and then the drug separates by breaking down from monomers. Then, the polymer of the drug links inside the micelle, followed by a diffusional discharge from the micelle core nanocarrier (Figure 3) [25,33]. In cancer therapy, polymeric micelles, rather than lipid micelles, have been used in several studies as optimal delivery vehicles for different therapeutic agents. Polymeric micelle nanocarriers reflect a particular interest in drug delivery applications because of their versatile characteristics, such as low cost, ease of synthesis, ease of conjugation of functional groups, biocompatibility, and biodegradability, among other characteristics [4,6,81].

With their ability to reduce MDR and increase the efficacy of co-delivery systems, these nanocarriers are being investigated more extensively [69,77]. Lv et al. have developed a new polymeric micelle to co-deliver DOX and curcumin (CUR) for MCF-7/ADR cells [14]. MCF-7/ADR cells have been frequently used in cancer research as a multidrug-resistant breast cancer cell type [81,82]. Lv et al. have developed a poly-(ethylene glycol)-block-poly-(lactide) (PEG2k-PLA5k) polymeric micelle and demonstrated an increase in the therapeutic efficacy of the co-delivery systems against MCF-7/ADR cells [14,83]. The approach used by Carvalho et al. demonstrated that the co-delivery system could generate increased tumor accumulation and good anti-tumor effects in MCF-7/ADR cells compared to single drug delivery using this method [65]. For the same drug combination of DOX and CUR, Zhang et al. developed methoxy poly (ethylene glycol) poly(caprolactone) micelles [26,81,84]. The latter co-delivery system sustained a suitable plasma concentration of active drugs by extending blood circulation and possessed slow-release characteristics [83]. Lin et al. created a multi-functional block copolymer micelle for the co-delivery of paclitaxel (PTX) and siRNA, a combination of an anti-cancer drug and a gene [81,85]. The latter micelle is unique in its ability to selectively deliver a combination of therapeutic agents. It has several important characteristics, including self-assembly, passive tumor targeting, and amplified cell death [86,87]. Furthermore, Wang et al. developed a new carrier for in vitro studies made up of PEG micelles and poly(e-caprolactone) (PCL) for the co-delivery of both PTX and an MDR reversal drug, commercially known as tacrolimus [74,88]. PEG-PCL micelles were found to reduce MDR effects by promoting apoptosis in human ovarian cancer cells by directly stimulating the apoptosis signaling system [14,21,84,88].

#### 2.2.4. Cubosome Nanoparticles

Cubosomes are self-assembled liquid crystalline particles, specifically created from the lipid cubic phase and stabilized by an outer corona, which is polymer based and can be employed for targeting [88]. A single lipid bilayer generates a continuous periodic membrane lattice structure with holes generated by two interlaced water channels in lipid cubic phases. The latter microstructure gives the cubosome distinctive features of practical significance, such as stability under different physiological settings. Cubosomes are considered a unique discovery that traverses the areas of three-dimensional geometry, organic membranes, and digestive processes [89]. The structure has a substantially larger membrane surface area than liposomes for loading membrane proteins and small molecule drugs. The cubic phases have generated a lot of interest because their unique architecture is medically friendly and capable of regulating the release of drugs, proteins, and other solubilized active components [90]. Cubosomes have different forms, in bulk or as nanoparticles. The latter has extensive potential applications ranging from drug delivery to membrane bioreactors, artificial cells, and biosensors.

Most studies on loaded cubosomes have been of proteins or small molecule drugs integrated inside the lipid membrane, with the use of single or binary lipid compositions based on monoolein or phytantriol. There have been multiple studies of cubosomes being loaded with small molecule drugs, including chemotherapeutic drugs for cancer treatment [91]. For small molecule chemotherapeutic drugs, studies show encapsulation effectiveness that ranges from 71 to 103 percent [90,92]. The capacity to create improved encapsulation and delivery vectors is critical to the success of gene technologies [92]. Cubosomes, when compared to a liposomal equivalent, contain a greater amount of siRNA. Notably, the cubosome membranes contain inherent fusogenic characteristics that encourage endosomal escape. Despite their considerable potential, typical cubosome formation approaches result in particle sizes that are too big to meet the advanced standards of delivery vectors. Therefore, Kim et al. utilized a microfluidic nanomanufacturing system to create cubosomes and siRNA-loaded cubosomes, known as cuboplexes [93]. Kim et al. successfully enabled the synthesis of tiny cubosomes and cuboplexes (75 nm) that surpassed the performance of commercially available delivery vectors, such as liposome-based arrangements, by using cryogenic TEM and small angle X-ray scattering through microfluidic devices [91,94]. Drug delivery, and specifically the co-delivery of multiple drugs, is now the most important biomedical use of cubosomes, since several of the fundamental elements have previously been approved for medical use. Since several drugs and therapeutic agents have limited solubility and permeability, lipid carriers are an appealing choice for drug delivery. While tremendous progress has been achieved in the past five years, there are currently no FDA-approved cubosome articulations for theragnostic purposes on the market; however, initial in vivo studies appear encouraging [88].

#### 2.2.5. Polymersomes

Polymersomes are self-assembled artificial vesicles that are empty at the center; thus, they have a structural pattern that is comparable to that of polymer liposomes [12]. Polymersomes are constructed from synthetic amphiphilic block copolymers such that hydrophilic molecules are in the aqueous solution-filled core, whereas the hydrophobic molecules are located within the membrane. Polymersomes are considered to have one of the most intriguing architectures for drug delivery systems because of their membrane resiliency [4,68,85]. These polymersomes can readily release drugs from external stimuli, such as ultrasound, light, and magnetic fields. For the targeted drug delivery system against the A549 cell line in lung cancer, polymersome nanoparticles were designed to load DOX and RNA aptamer, an epithelial cell adhesion molecule [30,95]. The polymersome was made up of FDA-approved polyethylene glycol and poly(lactic-co-glycolic) acid copolymer for the regulated drug delivery in A549 cells [21,27]. The targeted nanocarrier demonstrated low cytotoxicity through in vitro and in vivo assays. In addition, the targeted nanocarrier improved therapeutic effectiveness and had a higher inhibition of cancer cell proliferation when compared to non-targeted single drug delivery [64,74,95]. Table 2 presents selected clinically approved and marketed nano-drugs for cancer therapy.

Currently, significant attention has been directed to polymeric nanoparticles as innovative carriers for the majority of co-delivery systems [14,96]. The ability of the polymeric nanoparticles to carry hydrophobic and hydrophilic drugs together, as well as their beneficial ability to adequately control drug release, has made them favorable carriers [20,69]. In addition, polymeric nanocarriers possess advantageous characteristics, such as minimal systemic toxicity, high stability, and long circulation time, all of which help them accumulate in the tumor microenvironment. There have been several research and review papers published on cancer treatment by the co-delivery of different chemotherapeutic drugs via polymeric nanoparticles [20,23,30,95].

**Table 2:** Clinically approved and marketed nano-drugs for cancer.

Nanomaterial Type	Common Trade Name	Composition	Delivery	Indication	Approval Date	Company	Reference
Lipid-based nanoparticles	Doxil^®^/Caelyx^®^	PEGylated liposomal doxorubicin	Non-targeted delivery Immunoevasion	Metastatic ovarian, breast cancer, multiple myeloma, HIV-associated Kaposi’s sarcoma (KS)	FDA—1995 EMA—1996	Orthobiotech/Schering-Plough Canada Inc. Janssen-Cilag, Europe	[97,98,99]
	Lipodox^®^				FDA—2013	Sun Pharmaceutical Industries Ltd. (SPIL)	[97,100]
	DepoCyt^®^	Liposomal cytarabine	Non-targeted delivery	Lymphomatous meningitis	FDA—1999	Skye Pharma, Enzon	[77]
	DaunoXome^®^	Liposomal daunorubicin	Non-targeted delivery	HIV-associated Kaposi’s sarcoma (KS)	FDA—1996	Galen Ltd., USA/Gilead Science, Inc., Ireland	[78,101]
	Onivyde^®^	PEGylated liposomal irinotecan	Non-targeted delivery Immunoevasion	Metastatic pancreatic cancer	FDA—2015	Merrimack Pharmaceuticals Inc., Massachusetts, USA	[99,102]
Lipid-based nanoparticles	Myocet^®^	Non-PEGylated liposomal doxorubicin	Non-targeted delivery	Breast cancer	EMA—2000 (Approved in Europe and Canada)	Enzon Pharmaceuticals for Cephalon in Europe Elan Pharmaceuticals/Sopherion Therapeutics in Canada	[103,104,105]
	Mepact^®^	Liposomal mifamurtide	Non-targeted delivery	Osteogenic sarcoma	EMA—2009 (Approved in Europe)	Takeda France SAS	[101,106]
	Marqibo^®^	Liposomal vincristine sulfate	Non-targeted delivery Sustained Release	Acute lymphoblastic leukemia	FDA—2012	Talon Therapeutic, Inc., California, USA	[102]
Lipid-based nanoparticles	Lipusu^®^	Liposomal paclitaxel	Non-targeted delivery Sustained Release	Breast cancer, NSCLC, ovarian cancer	Approved in China—2006	Luye Pharma Group	[107,108,109]
	Vyxeos^®^	Liposomal daunorubicin and cytarabine	Combinatorial delivery	Acute myeloid leukemia	FDA—2017 EMA—2018	Jazz Pharmaceutics, Inc.	[106,110]
Polymer-based nanoparticle	Genexol-PM^®^	Paclitaxel micellar	Sustained Release	Breast, ovarian, gastric cancer, and NSCLC	Approved in South Korea—2007	Samyang, Seongnam, South Korea	[111,112]
	Eligard^®^	Leuprolide acetate and polymer	Non-targeted delivery	Prostate cancer	FDA—2002	Tolmar Pharmaceuticals Inc.	[113,114]
Protein- drug conjugate	Pazenir^®^	Paclitaxel	Non-targeted delivery	Metastatic breast cancer, metastatic adenocarcinoma of the pancreas, NSCLC	EMA—2019	Ratiopharm GmbH San Francisco, CA, USA	[107]
	Oncaspar^®^	PEGylated L-asparaginase conjugate	Non-targeted delivery	Acute lymphocytic leukemia	FDA—1994	Enzon Pharmaceuticals Inc.	[114,115,116]
Protein nanoparticle	Abraxane^®^	Albumin-bound paclitaxel	Non-targeted delivery Sustained Release	Metastatic breast cancer Lung cancer, and NSCLC	FDA—2005 EMA—2008 FDA—2012	Abraxis Bioscience, AstraZeneca	[117,118,119]
		Nab-paclitaxel in combination with gemcitabine		Metastatic pancreatic adenocarcinoma	FDA—2013	Celgene Pharmaceutical Co. Ltd.	
Inorganic nanoparticle	Hensify^®^	Hafnium oxide nanoparticles	Non-targeted delivery Radiation-activated	Locally advanced squamous cell carcinoma	EMA—2019	Nanobiotix	[110]
	Nano-therm	Iron oxide (Fe_2_O_3_)	Hyperthermia	Glioblastoma, prostate, and pancreatic cancer.	FDA—2010 EMA—2013	Magforce	[102]

Abbreviations: FDA: Food and Drugs Administration; EMA: European Medicines Agency; NSCLC: non-small cell lung; PEG: polyethylene glycol.

## 3. Combination Treatment Strategies

In many circumstances, the aim of creating co-delivery systems is to combine different therapeutic agents to have multiple treatments to break through the biological barrier and halt the proliferation and growth of cancer cells [13,95]. The interplay of each component, the balance of underlying competing elements, and synergistic treatment options should all be considered when constructing effective co-delivery systems. All the latter elements must work together to ensure excellent overall therapeutic efficacy [38].

### 3.1. Co-Delivery of Chemotherapeutic Drugs

Chemotherapeutic agents are divided into three categories: anthracyclines, taxanes-based antineoplastic medicines, and DNA alkylating agents, which include platinum-based anti-tumor chemicals [19,25]. The latter chemotherapy drugs are categorized based on chemical properties, such as hydrophobicity, and their effect on tumor cells [63]. Despite their fundamental role in cancer treatment, many chemotherapeutic agents have been linked to adverse side effects and limited absorption. Furthermore, chemotherapeutic drugs tend to accumulate in the tissue due to their use in high concentrations, leading to MDR [68,120]. To reverse MDR, treating patients with multiple chemotherapeutic drugs or chemosensitizers at the same time are the most prevalent options [13,121,122].

DOX, a chemotherapeutic anthracycline, has been used clinically to treat several hematologic malignancies and solid tumors, including lung, breast, and pancreatic cancer [5,28]. However, using DOX as a mono-therapeutic treatment usually causes severe side effects in normal tissues, especially cardiotoxicity. The reason behind the cardiotoxicity induced by DOX is still unclear. However, it has been postulated that this side-effect is caused by the conversion of quinone into free radicals of half quinone, which in turn initiate a cascade of reactions leading to the production of reactive oxygen and nitrogen in the body [36,86,88]. To achieve a synergistic effect, overcome the MDR of DOX, and simultaneously tone down the cardiotoxicity, DOX is often used in combination with another chemotherapeutic drug. DOX is usually combined with Paclitaxel (PTX) in different types of nanoparticles; however, the best encapsulation is in a polymeric nanoparticle [84,88]. Wang et al. and Lv et al. designed core-shell methoxy polyethylene glycol-polylactic-co-glycolic acid (mPEG-PLGA) nanoparticles loaded with DOX and PTX [66,123]. Specifically, loading of DOX and PTX by dual emulsion method enhanced the anti-tumor efficacy and release of the drugs from the nanocarrier at the acidic tumor location [88,122,124]. The combination of DOX and PTX is better and more efficient than the single administration of either DOX or PTX at the same concentrations. DOX has a faster release rate than PTX, and the release of DOX facilitates the release of PTX. Therefore, the drugs showed a higher synergism in the co-delivery system at a molar ratio of 2:1, due to the difference in drug release rates [5,20,69].

Another study investigated an equivalent co-delivery system, that encapsulated hydrophobic irinotecan (CPT-11) and hydrophilic DOX [13,23]. For breast and brain cancer, especially, the latter drug delivery system decreased the action of topoisomerase I and II, resulting in a more effective treatment [25,77]. Another study experimented with a graft copolymer with side drug segments that form nanostructures utilizing a protein folding route to distribute camptothecin (CPT) and doxorubicin [19,125]. The assembly of this nanoparticle is driven by the hydrophobicity of the conjugated CPT. When the copolymer is subjected to water, it locks with DOX to produce monodisperse nanolayers that transport DOX and CPT together. In several tumor cell lines, these nanolayers resulted in strong synergistic action of the CPT and DOX [23,125,126,127]. In a lung cancer model, the in vivo study revealed that the nanolayers could accumulate at tumor locations and have significant anti-tumor action compared to single drug administration. Another combination system is DOX and verapamil (VER), specifically in pH-sensitive polymeric nanoparticles [13,14,128,129]. The polymeric nanoparticle is based on the co-polymer, methoxy-poly (ethylene glycol)2k-poly(e-caprolactone)4k-poly (glutamic acid)1k (mPEG2k-PCL4k-PGA1k-FA) [14,129]. In vitro anti-cancer studies showed that the co-delivery system could overcome multi-drug resistance, achieve high drug release efficiency, and improve anti-cancer impact [74,88]. A further summary of the different combinations of dual drug delivery systems is shown in Table 3.

### 3.2. Co-Delivery of Chemotherapeutic Drugs and Genes

Currently, the majority of anti-cancer efforts are focused on creating techniques that can block the efflux pump effects generated by long-term pharmacological therapy. Combining chemotherapeutic drugs with additional therapeutic genes, such as nucleic acids, is another promising option for a co-delivery system. This co-delivery system can also be referred to as gene therapy [40,95,128,138]. The latter is defined as releasing external normal genes into target cells to counterweigh faulty and aberrant genes. Therapeutic genes, such as microRNA (miRNA), small interfering RNA (siRNA), and plasmid DNA (pDNA), are critical in cell formation, assimilation, and apoptosis [69,128,139]. The most common delivery strategy is to adsorb the genes onto the nanoparticle’s surface and encapsulate chemotherapeutic drugs inside the chosen nanocarrier. The incentive behind the co-delivery of genes and chemotherapeutic drugs is to disrupt MDR signaling pathways [95,140]. This disruption helps overcome MDR in cancer cells while increasing the response to chemotherapeutic drugs like anthracyclines and other anti-cancer treatments [131,141].

Jie Liu et al. produced positive surface charge polyphosphoester-based micelles for lung cancer treatment. DOX and the tumor suppressor gene p53 combination were encapsulated inside these micelles [84]. In vitro investigations revealed that these micelles might successfully transfer the drug-gene combination into the A549 cell line; the A549 cell line is composed of hypotriploid alveolar basal epithelial cells [142,143,144]. Hao et al. revealed that experimental results showed that the latter cationic micelles have higher anti-tumor effects compared to single drug delivery [52,145]. Bhattarai et al. used a solvent evaporation method to create pH-responsive poly(2-(dimethylamino) ethyl methacrylate)-block-poly(2-(diisopropylamino) ethyl methacrylate) micelles [136]. The latter micelles were used to reverse MDR in lung cancer by co-delivering chemotherapeutic agents and siRNA [146]. The results of the experiment conveyed that nude mouse models carrying A549 tumor cells showed better anti-tumor activity [36,147,148]. Table 4 summarizes some relevant variations of drug-gene co-delivery systems and the corresponding nanocarriers for cancer therapy.

### 3.3. Co-Delivery of Multiple Genes

Substitutes to chemotherapy include co-delivery of gene agents with various RNA-based mechanisms via a single route of administration. Dual genetic material may have more substantial therapeutic benefits than single gene agent delivery, including different nucleic acids, such as pDNA, siRNA, shRNA, miRNA, etc. [14,158]. Co-delivery of the latter nucleic acids has the potential to guide and regulate many intracellular components, hence changing cellular activity and disease development. The mechanism of MDR in cancer treatment is closely connected to the overexpression of transporter proteins that release absorbed therapeutic agents [118]. As a result, many attempts and studies have been undertaken in the screening of different nano-formulations to investigate overcoming MDR through gene delivery. Some researchers have advocated the creation of nano-platforms for dual gene delivery, such as siRNA-siRNA, pDNA-siRNA, shRNA-siRNA, siRNA-miRNA, etc., to aid in the treatment of disorders linked with various gene dysregulations [14,159].

Dual gene delivery has more substantial therapeutic benefits than the delivery of a single gene agent, such as pDNA, siRNA, shRNA, or miRNA alone. In this regard, non-viral vector carriers arise as an option to permit intracellular co-delivery of nucleic acids [150]. Co-delivery of pDNA with siRNA, for example, can be used to extend silencing effects; although siRNA may be reduced to one specific gene, pDNA acts by overexpressing a tumor suppressor gene [129]. Tabernero et al. conducted the first-in-human clinical trials (phase I), in 2013, on the siRNA co-delivery method that used a lipid nanoparticle to transport two modified siRNAs [151,160]. One siRNA targeted anti-VEGF expression and the other suppressed kinesin spindle protein (KSP). KSP siRNA was employed to impair cell proliferation and cause cell death, while anti-VEGF siRNA was utilized to inhibit angiogenesis. The lipid nanoparticle was shown to be safe and tolerable after biweekly dosing [161,162]. The experiment, which included individuals aged 18 and up, demonstrated the formulation of the pharmacokinetics, RNAi mode of action, and clinical anticancer potential. The simultaneous administration of both genetic materials might cause a more successful combination treatment than the delivery of simply one nucleic acid [153,161].

Despite the progress made regarding various novel nanocarriers for gene therapy, the most distinguished non-viral vectors are only capable of transporting a single gene agent (pDNA or shRNA). Consequently, developing dual gene delivery nanocarriers, able to load various nucleic acids efficiently, remains a difficulty in gene therapy [153,163]. Table 5 summarizes the co-delivery systems utilizing nanocarriers for dual nucleic acid delivery that can be categorized based on their base materials, which include polymer-based, lipid-based, and inorganic-based nanocarriers [14,164].

## 4. Limitations and Challenges

Although developments have been made with co-delivery systems accompanied by nanotechnology, there are still a few issues to consider when designing a synergistic drug delivery system. The benefits and drawbacks of co-delivery systems are summarized in Table 6 [22]. The key benefit of co-delivery systems is the possibility of synergistic effects, due to coordinated biodistribution and the combination of different therapeutic agents. Most significantly, increasing drug concentrations within the nanoparticle, controlling drug release progressively, and encapsulating drugs with different moieties and physicochemical properties in vivo, present critical challenges in the design of drug-drug or drug-gene-loaded nanocarriers [72,95,171].

Although numerous nanomedicines have been approved for different medical purposes, global monitoring ethics and policies remain unsubstantiated and underdeveloped, thereby limiting the possibility of sustainably producing nanomedicines that meet Good Manufacturing Practice (GMP) to become prime therapeutic agents. In addition, numerous hurdles and issues exist in the clinical translation of these systems into real-life practical treatment plans [162]. The primary issues involved with nanomedicine regulation relate to the intrinsic features of nanomedicines. Specifically, the different pharmacodynamic and pharmacokinetic characteristics of nanoparticles when compared to their constituent ingredients and loads. There is also a wide gap between animal and human trials that, in turn, hinders advancement in nanomedicine translation. Animal models lack the capability of reproducing all characteristics of human malignancies, being a major contributing factor to the disparity between therapeutic effectiveness detected in preclinical studies and the absence of encouraging clinical results [172,173]. Furthermore, it is difficult to anticipate the possible harmful effects of nanoparticles in vivo since several preclinical investigations are performed on immunodeficient animal models, and most patients have fully intact immune systems. Moreover, there are considerable disparities in relative size between the accumulation of cancer cells implanted in animal models compared to cancer tissues that form spontaneously in people. For instance, some studies and clinical research reported that the EPR effect works in rats but not in humans. These incompatibilities stem from several factors, such as a lack of knowledge in terms of the physical, biological, and pathological differences between animal models and humans [164,174]. Clinical translation is also hampered by factors other than species differences. For instance, patient heterogeneity and the unexplored variability in the disease’s biological foundation within patients impact the usefulness, dispersion, functionality, and uptake of nanoparticles in the body [163]. The latter is due, in part, to hindrance in the efficacy of nanoparticles as drug carriers and restriction in the number of approved nanomedicines offered to patients.

Furthermore, the co-delivery of drugs with different-sized nanoparticles is relatively difficult. Utilizing nanoparticles smaller than 200 nm is efficient in extremely porous murine tumor microenvironments [23,175]. After various in vivo testing and analysis, Cabral et al. stated that only 30 nm polymeric micelles could penetrate weakly permeable pancreatic cancer, but polymeric micelles with diameters larger than 50 nm are too large for membrane penetration [23,176,177]. As a result, the desired particle size has reduced intensely during the last decade from 200 nm to 50 nm. Since a 50 nm nanoparticle has 64 times fewer encapsulated drugs than a 200 nm nanoparticle, delivering 64 times more of the smaller particles is essential to provide the same drug dosage. This challenge regarding nanoparticle size in a co-delivery system can also be referred to as a “Particle Size Dilemma” [123,125,177]. In addition, the high cytotoxic levels of nanocarriers, the need for efficient therapeutic agent ratios and for sequential drug release patterns of the respective therapeutic agents are significant impediments to nano carrier-based co-delivery systems [112]. As a result, few nanomedicines are suggested as a primary therapy alternative and several demonstrated benefits in only a slight proportion of patients. Studies have been carried out to improve the toxicity of current nanocarriers and to generate new nanocarriers that are less hazardous and exhibit more rapid and efficient clearance [161]. Along with the various nanoparticles presented, inorganic-based nanocarriers require comprehensive toxicity research for future clinical exploration. However, polymeric nanoparticles, specifically biodegradable polymeric nanoparticles, are biocompatible and showed less cytotoxicity compared to inorganic-based nanocarriers [14,69]. Nano-based co-delivery systems are vital, not just in terms of development, but also in terms of proper evaluation. Tackling the latter limitations and challenges will all aid in validating co-delivery systems and improving the synergistic effects of co-delivered therapeutic agents [20,77].

## 5. Conclusions and Future Perspectives

Despite the substantial progress made in the process of the single administration of therapeutic drugs (monotherapy) in recent years, it is a combination therapy that has achieved rapid development. However, combination therapy is unquestionably more difficult than monotherapy; given that the co-delivery system loads two or more anti-cancer drugs with different physicochemical and pharmacological properties [66,77,125]. Co-delivery systems make it feasible to combine different therapeutic agents, whether chemotherapeutics, genes or other active ingredients, and target them simultaneously to the neoplastic site. Combination therapy can only be realized with the help of nanotechnology. Nanotechnology has been used to safeguard and securely release therapeutic agents in order to ensure effective intracellular delivery. The first aspect of efficient co-delivery systems is concerned with the design and construction of nanocarriers, whereas the second part is concerned with the combined application of different therapeutic agents [84]. Through co-delivery systems, synergies between multiple therapeutic agents or techniques can offer good therapeutic outcomes. For instance, the combination of chemotherapeutic agents was shown to exhibit the potential to overcome MDR and achieve notable therapeutic efficacy [73]. Moreover, co-delivery systems accompanied by nanocarriers synergistically repressed the proliferation of cancer cells compared with monotherapy [77,143]. It is important to consider, and thoroughly investigate, the interactions between the loaded drugs and carriers, as well as the stability of such delivery methods [131]. The design and use of nanocarriers capable of carrying two or more therapeutic agents with distinct characteristics is still an emerging field, due to its complexities [168]. Therefore, further in vivo and clinical investigations are needed before such formulations can make it from bench to bedside.

The reviewed nanoscale drug delivery systems included polymeric micelles, polymersomes, liposomes, mesoporous silica nanoparticles, lipid nano-emulsions, gold nanoparticles, iron-oxide nanoparticles, etc. In the design process, the latter nanocarriers have been adjusted to encapsulate two or more drugs or genes [20,128]. Furthermore, a multidrug-loaded nanocarrier facilitates the simultaneous release of two or more encapsulated therapeutic agents. Within the nanocarriers, drug-drug interactions are reduced, and the pharmacokinetic profile of loaded therapeutic agents is improved using such nano-drug delivery technology. As a result, future research in this field should concentrate on developing and addressing several aspects, including effectiveness in cancer cell targeting, loading capability, unexpected degradation in vivo, release kinetics, and biocompatibility. The possible restraint of each component of co-delivery systems, including carriers and drugs, should be carefully considered. A careful balance between each component necessitates accurate regulation [128,178].

In this review, the current standpoint, emerging trends of co-delivery systems and the most frequently utilized nanomaterials, were examined. In addition, a compilation of current breakthroughs in the field of co-delivery employing lipid, polymeric, and inorganic-based nanoparticles, published in the previous 10 years, was undertaken [13]. As the first stages of clinical trials and research in this field progress, the co-delivery of multiple therapeutic drugs will provide motivation and inspiration for further growth and advancement in terms of reaching further human trials. Unlimited combinations of active agents can now be realized with the currently available technologies for simultaneous targeting and delivery, making co-delivery systems promising therapeutic platforms for cancer therapy. Extensive studies are required to commercialize co-delivery systems, which are expected to ultimately surpass clinical trials to extend cancer patients’ lives and enhance the quality of their lives.

## Figures and Tables

**Table 3:** Co-delivery of drug-drug by various nanocarrier systems for cancer therapy.

Delivery System	Drug-Drug	Cell-Line	Indication	Reference
π–π stacked dual anti-cancer drug combination with an actively targeted, pH- and reduction-sensitive polymer micellar platform	DOX and HCPT (10-hydroxycamptothecin)	MCF-7/ADR	Breast cancer	[120,130]
Inorganic nanoparticle Graphene oxide functionalized with a carboxyl group (GO-COOH)	DOX and Curcumin (CUR)	AGS, PC3, A2780, and HFF	Gastric, prostate, and ovarian cancer	[121,131]
Transferrin conjugated Liposomes	DOX and Verapamil (VER)	K562	Leukemia	[72]
Amphiphilic methoxy polyethylene glycol-polylactic-co-glycolic acid (mPEG-PLGA) nanoparticles	DOX and Paclitaxel (PTX)	A549, SK-MEL-3	Non-small lung cancer and melanoma	[13,88]
pH-responsive Metallo-supramolecular nanogel (SNG)	DOX and Tetraphenylporphyrin zinc	HepG2, A431	Liver cancer, and epidermoid carcinoma	[122,132]
Liposome	PTX and trichosanthin (TCS)	A549	Lung cancer	[123,133]
Hyaluronic acid nanogel (HANG)	Cisplatin (CDDP) and DOX	K7 cells (mouse osteosarcoma)	Osteosarcoma	[124,134]
pH-sensitive biomimetic nanoparticles (MNPs)	Temozolomide (TMZ) and Cisplatin (CDDP)	U87MG	Glioblastoma (GBM)	[125,135]
ApoE-functionalized liposomes based on artesunate-phosphatidylcholine (ARTPC) encapsulated with temozolomide (ApoE-ARTPC@TMZ)	Artesunate (ART) and temozolomide (TMZ)	U251-TR	Glioblastoma (GBM)	[126,136]
pH-sensitive ApoE peptide decorated biomimetic nanomedicine (ABNM@TMZ/OTX)	Temozolomide (TMZ) and OTX015 (OTX)	GL261 GBM	Glioblastoma multiforme (GBM)	[127,137]

**Table 4:** Co-delivery of small molecule drugs and therapeutic genes for cancer therapy.

Nanocarrier	Delivery System	Drug-Gene	Cell-Line	Indication	Reference
Mesoporous silica nanoparticle (MSN)	Mesoporous silica nanoparticles modified with polyethyleneimine MSNP-PEI-PEG	DOX—ABCB1 (P-gp)	KB-V1	Oral squamous carcinoma	[50]
Micelle	Triblock copolymer functionalized with folic acid: PEG-PCL-PEI	DOX—(P-gp) siRNA	MCF-7, ADR	Breast cancer	[149]
	A reduction and pH dual sensitive ternary block copolymer PEG-PAsp(AED)-PDPA	DOX—BCL-2 siRNA	SKOV3	Ovarian cancer	[150]
Self-assembled cationic micelle	Folate conjugated ternary copolymer FA-PEG-PEI-PCL	DOX—BCL-2 siRNA	SKOV3	Ovarian cancer	[151]
Polymeric micelle	Targeted multi-functional polymeric micelle (TMPM): triblock copolymer PCL-PEG-PHIS	Paclitaxel—VEGF siRNA (siVEGF)	HUVECs, MCF-7	Breast Cancer	[152]
Polymer-based nanomaterials	Hypoxia-sensitive PEG-azobenzene-PEI-DOPE (PAPD) nanoparticles	DOX—ABCB1 siRNA	A2780 ADR, MCF7 ADR	Ovarian cancer and breast cancer	[153]
	Chitosan-based pH-responsive polymeric prodrug vector GA-CS-PEI-HBA-DOX	DOX—BCL-2 siRNA	HUVEC, HepG2	Liver cancer	[154]
Cationic liposome	PEGylated liposomes	Docetaxel—BCL2 siRNA	A549, H226	Lung cancer	[155]
	Thermosensitive magnetic cationic liposomes (TSMCL)	DOX—SATB1-shRNA	MKN-28	Gastric adenocarcinoma	[156]
Dendrimer	PAMAM-PEG-T7	Doxorubicin—plasmid pORF-hTRAIL	Bel-7402	Liver cancer	[157]

Abbreviations: DOX: Doxorubicin; PEG: poly(ethylene glycol); PEI: poly(ethylene imine); PCL: poly(ε-caprolactone); PDPA: poly(2-(diisopropyl amino)ethyl methacrylate); PAsp(AED): poly(N-(2,2′-dithiobis(ethylamine)) aspartamide); PEG: poly(ethylene glycol); PHIS: poly(L-histidine); GA: Glycyrrhetinic acid; CS: Chitosan; HBA: hydrazinobenzoic acid; T7: a transferrin receptor-specific peptide; ABCB1: ATP binding cassette subfamily B member 1; BCL2: BCL2 apoptosis regulator; VEGF: vascular endothelial growth factor group.

**Table 5:** Nanocarrier systems for dual delivery of nucleic acids for cancer treatment.

Nanocarrier	Delivery System	Active Agents	Cell-Line	Indication	Reference
Polymer-based	Polyethylenimine–poly(L-serine) (PEI–PSer)	Bcl-2-siRNA and pKH-rev-casp-3 (siRNA and pDNA)	HeLa/293T-GFP	Cervical carcinoma, kidney cancer	[165]
	Poly(DL-lactide-co-glycolide acid) (PLGA) nanoparticles and poly-L-lysine (PLL) as a complexing reagent (PLGA NPs)	MDR1-siRNA and Bcl-2-siRNA (siRNA and siRNA)	SKOV-3/A2780-CP20	Ovarian cancer	[166]
	poly(l-lysine) (PLL)—oligomeric sulfonamides (OSA)	luc-siRNA and pLuc (siRNA and pDNA)	HEK293 (human embryonic kidney cell line)	Renal cancer	[167]
Lipid-based	Lipid nanoparticle (LNP)	anti-VEGF-siRNA and KSP-siRNA	SCID/Hep3B	Hepatocellular carcinoma	[161]
	LPH (liposome-polycation-hyaluronic acid (HA)) nanoparticle formulation modified with tumor-targeting single-chain antibody fragment (scFv)	siRNA and miRNA (c-myc/MDM2/VEGF-siRNA and miR-34a)	B16F10	Lung cancer	[168]
	anti-EGFR aptamer coupled cationic lipid nanocarriers incorporated with hydrophobic quantum dots (QDs) QD-lipid nanocarriers (QLs)	Quantum dots (QDs) and siRNA-siRNA (Bcl-2-siRNA and PKCl-siRNA	MDA-MB-231/MDA-MD-453	Breast cancer	[93]
Inorganic-based	pH-sensitive carbonate apatite (CO3Ap) nanoparticles	siRNA-siRNA (ABCG2-siRNA and ABCB1-siRNA	MCF-7	Breast cancer	[169]
	Polymer-Coated Gold Nanoparticles (MAu-P-D-SS37-siRNA-447)	anti-eGFP siRNA and pEGFP-N1, pDsRed-Max-N1 (siRNA-pDNA)	GBM319	Brain cancer	[170]

**Table 6:** Summary of the benefits and drawbacks of co-delivery systems.

Advantages	Disadvantages
Reducing cytotoxicity	Higher risk of drug interactions
Enhancing synergistic effects	Difficulty in reducing particle size
Improving the quality of life for patients	Challenge in coordinated drug release
Synchronized biodistribution	Complicated preparation and high cost
Reducing the likelihood of MDR	Antagonistic effects

## Data Availability

Not applicable.
